# The Chromodomain of LIKE HETEROCHROMATIN PROTEIN 1 Is Essential for H3K27me3 Binding and Function during Arabidopsis Development

**DOI:** 10.1371/journal.pone.0005335

**Published:** 2009-04-28

**Authors:** Vivien Exner, Ernst Aichinger, Huan Shu, Thomas Wildhaber, Pietro Alfarano, Amedeo Caflisch, Wilhelm Gruissem, Claudia Köhler, Lars Hennig

**Affiliations:** 1 Department of Biology & Zurich-Basel Plant Science Center, ETH Zurich, Zurich, Switzerland; 2 Department of Biochemistry, University of Zurich, Zurich, Switzerland; Cairo University, Egypt

## Abstract

Polycomb group (PcG) proteins are essential to maintain gene expression patterns during development. Transcriptional repression by PcG proteins involves trimethylation of H3K27 (H3K27me3) by Polycomb Repressive Complex 2 (PRC2) in animals and plants. PRC1 binds to H3K27me3 and is required for transcriptional repression in animals, but in plants PRC1-like activities have remained elusive. One candidate protein that could be involved in PRC1-like functions in plants is LIKE HETEROCHROMATIN PROTEIN 1 (LHP1), because LHP1 associates with genes marked by H3K27me3 *in vivo* and has a chromodomain that binds H3K27me3 *in vitro*. Here, we show that disruption of the chromodomain of *Arabidopsis thaliana* LHP1 abolishes H3K27me3 recognition, releases gene silencing and causes similar phenotypic alterations as transcriptional *lhp1* null mutants. Therefore, binding to H3K27me3 is essential for LHP1 protein function.

## Introduction

Polycomb group (PcG) proteins maintain gene expression patterns during development in animals and plants by establishing a cellular memory system for transcriptional repression [1]. Although many functional details of PcG proteins remain unknown, current models suggest that repression involves trimethylation of histone H3 lysine 27 (H3K27me3) by Polycomb repressive complex 2 (PRC2). In insects and mammals, H3K27me3 assists in the recruitment of PRC1 [2]. Binding of PRC1 to H3K27me3 is mediated by the chromodomain of the PRC1 subunit Polycomb (Pc) [3]. Metazoan PRC1 complexes catalyze H2A monoubiquitylation via their RING-domain subunits and are needed for stable repression of PcG target genes [2]. Although the PcG system is present in plants and PRC2 homologs have similar functions, no clear plant PRC1 homologs have been identified [1]. Proteins that may have PRC1-like functions in plants include EMBRYONIC FLOWER 1, VERNALIZATION 1, LIKE HETEROCHROMATIN PROTEIN 1 (LHP1) and RAWUL-proteins [4]–[7].

The gene for *Arabidopsis thaliana* LHP1 was first found in screens for mutants with altered leaf glucosinolate levels and named *TU8* [8], [9] as well as in screens for inflorescence meristem function and named *TERMINAL FLOWER 2* [10], [11]. In addition, LHP1 was identified as a homolog of metazoan HETEROCHROMATIN PROTEIN1 (HP1) [12]. Similar to HP1, LHP1 contains a chromodomain and a chromo shadow domain [11], [12]. Unlike HP1, however, LHP1 is usually localized in euchromatin and is needed for maintenance of gene silencing in euchromatin but not in heterochromatin [13], [14]. Finally, LHP1 can bind to H3K27me3 *in vitro* and associates with genes marked by H3K27me3 *in vivo* [15], [16]. Homologs of the animal PRC1 core component RING1 have recently been identified in Arabidopsis, and binding of AtRING1A to LHP1 suggests similar structure and function of plant and animals PRC1 complexes [17].

Together, the model has emerged that LHP1 binds to PcG target loci that have been trimethylated at H3K27 by PRC2 to establish persistent transcriptional repression. We tested this hypothesis using a LHP1 mutant with a defective chromodomain. In agreement with predictions from structural homology-based modeling, LHP1 with the mutated chromodomain had strongly reduced binding to H3K27me3 *in vitro*. Furthermore, recruitment to target genes and intra-nuclear localization of mutated LHP1 was greatly impaired *in vivo*. Because the phenotype of this new *lhp1* allele is very similar to an *lhp1* null allele, we conclude that chromodomain-mediated binding of LHP1 to H3K27me3 is essential for LHP1 function. These results support the model that LHP1 has a PRC1-like function in plants.

## Results

### An LHP1 mutant protein with a defective chromodomain

**Figure 1:**
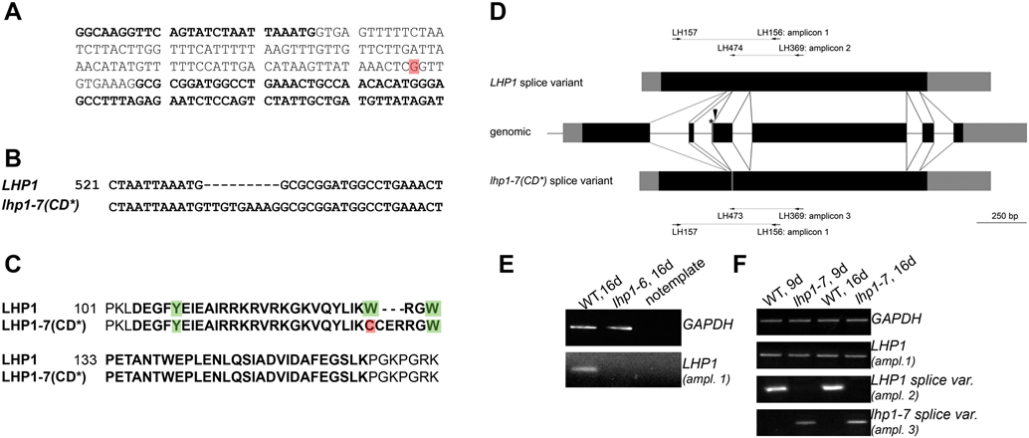
A novel *lhp1* allele. (A) Part of the genomic sequence of wild-type *LHP1*; exons are marked in bold. The EMS allele *lhp1-7* has a G to A transition in the second intron (position marked in red). (B) The point mutation in *lhp1-7* creates a new splice site. The critical region of the alignment of wild-type and mutant *LHP1* cDNAs is shown. (C) The lhp1-7 mutant protein has a defect in the chromodomain. The critical region of the alignment of the wild-type and mutant LHP1 proteins is shown. The chromodomain is shown in bold; the aromatic cage residues are marked in green and the cysteine that substitutes one of them in the mutant protein is marked in red. (D) The structure of wild-type and mutant *LHP1* transcripts and primers for PCR amplicons. Black boxes, grey boxes and lines represent exons, untranslated regions and introns, respectively. The asterisk marks the position of the point mutation in *lhp1-7* and the additional exon inclusion is shown in dark grey. The arrow marks the position of the T-DNA insertion in *lhp1-6*. Note that amplicon 1 is not specific for either splice variant while amplicons 2 and 3 are specific for the wild-type and mutant splice variants, respectively. For primer sequences see Table 3. (E) *lhp1-6* is a transcriptional null mutant. (F) *lhp1-7* expresses the mutant splice variant at the same level as the wild-type expresses *LHP1*. RNA in (E, F) was isolated from seedlings grown under long day conditions for 9d or 16d.

The new *lhp1-7* allele was discovered in a suppressor screen of a late flowering transgenic line with reduced MSI1 function (*msi1-tap1*; [18]). For details of the mutant screen see Materials and Methods. Sequencing of the *LHP1* locus and the *LHP1* cDNA revealed a newly created splice site that led to the presence of nine additional nucleotides at the junction of exons two and three in the processed *lhp1-7* transcript (Fig. 1A, B). This results in three additional amino acids (Cys-Glu-Arg) in the chromodomain adjacent to the conserved tryptophan 129, which is changed into a cysteine (Fig. 1C). The *lhp1-7* allele was introduced into the Columbia wild-type by backcrossing, and all further experiments were performed with *lhp1-7* in the wild-type background unless otherwise specified. We compared *lhp1-7* to the *lhp1-6* null allele, which we isolated previously from the SALK T-DNA insertion collection (line SALK_011762). While no *LHP1* transcript was detected in the *lhp1-6* T-DNA insertion mutant (Fig. 1D, E), *LHP1* transcript levels in *lhp1-7* were similar to those in wild-type (Fig. 1F). However, in *lhp1-7* only the mutant but not the wild-type splice variant was detected (Fig. 1F), suggesting that *lhp1-7* produces no or only very little wild-type protein. We discovered *lhp1-7* in a screen for suppressors of reduced MSI1 function, but the potential link between LHP1 and the histone binding WD40 repeat protein MSI1 will be discussed elsewhere (for a review about MSI1-like proteins see [19]). Here, we used the new *lhp1-7* allele to probe LHP1 function *in vitro* and *in vivo*.

**Figure 2:**
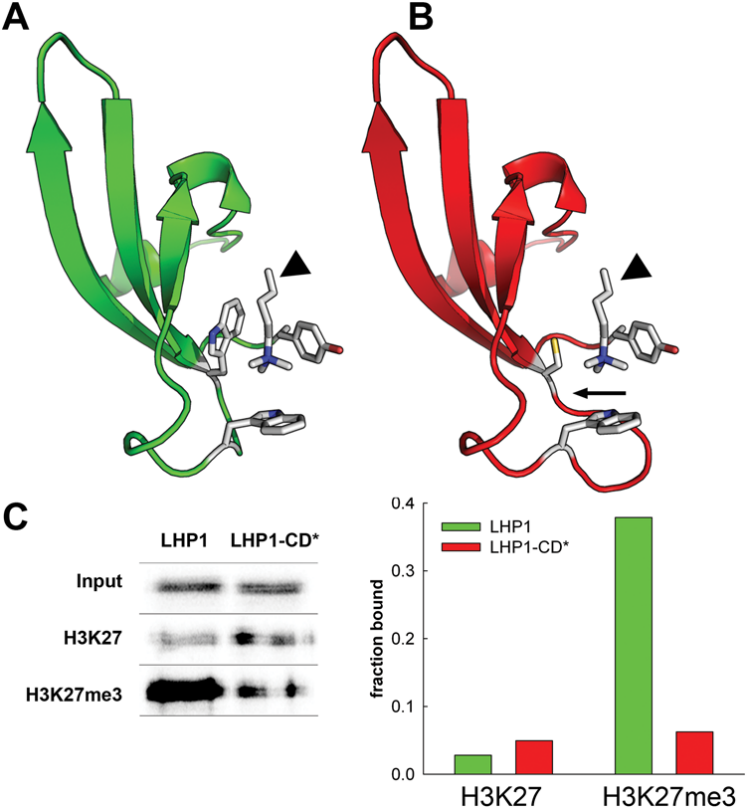
lhp1-7(CD*) encodes an LHP1 mutant protein with a defective chromodomain. (A) Structural model of the LHP1 chromodomain based on homology modeling using the coordinates of the Drosophila Pc chromodomain complexed with an H3K27me3 peptide [3]. (B) Structural model of the LHP1-CD* chromodomain, which is encoded by lhp1-7(CD*). The arrow indicates the mutated region in LHP1-CD*. The position of the trimethylated lysine side chain (arrow heads) in (A) and (B) was derived from the template crystal structure. (C) Peptide-binding pull-down assay for wild-type LHP1 and LHP1-CD* (left) and quantification (right).

The HP1 and Pc chromodomains have binding cavities formed by three aromatic residues to accommodate methylated lysines of H3 histone tails [3], [20], [21]. Homology-based modeling revealed that similar to HP1 and Pc, the chromodomain of LHP1 has the potential to form a binding cage containing three aromatic residues (Fig. 2A). Because one of the three aromatic residues, tryptophan 129, was changed to a cysteine in the chromodomain of lhp1-7, it is likely that this protein cannot form the typical binding cage and will be called LHP1-CD* (Fig. 2B). To more easily distinguish between the *lhp1-6* null allele and the *lhp1-7* mutant, we will refer to these alleles as *lhp1-6 (null)* and lhp1-7 (CD*). Calculation of interaction energies suggested that LHP1-CD* has reduced affinity to trimethylated and unmethylated lysine residues (Table 1).

**Table 1:** Intermolecular energy values in Kcal/mol calculated by CHARMM upon minimization using a distance dependent dielectric function.

	Van der Waals	Electrostatic	Total
LHP1-CD/H3K27me3	−25.0	−3.9	−28.9
LHP1-CD*/H3K27me3	−22.5	−3.0	−25.5
LHP1-CD/H3K27	−19.5	−3.3	−22.8
LHP1-CD*/H3K27	−15.9	−2.5	−18.4

Next, we tested whether binding to H3K27me3 was indeed affected by the lhp1-7 (CD*) mutation. Similar to previously reported results, wild-type LHP1 bound strongly to the H3K27me3 peptide *in vitro*, but LHP1-CD* binding to H3K27me3 was significantly reduced and similar to the binding to unmethylated H3K27 (Fig. 2C). The reduced binding affinity to H3K27me3 *in vitro* suggests that LHP1-CD* could have compromised activity *in vivo*.

### The LHP1 chromodomain is required for correct sub-nuclear localization and binding to target genes

**Figure 3:**
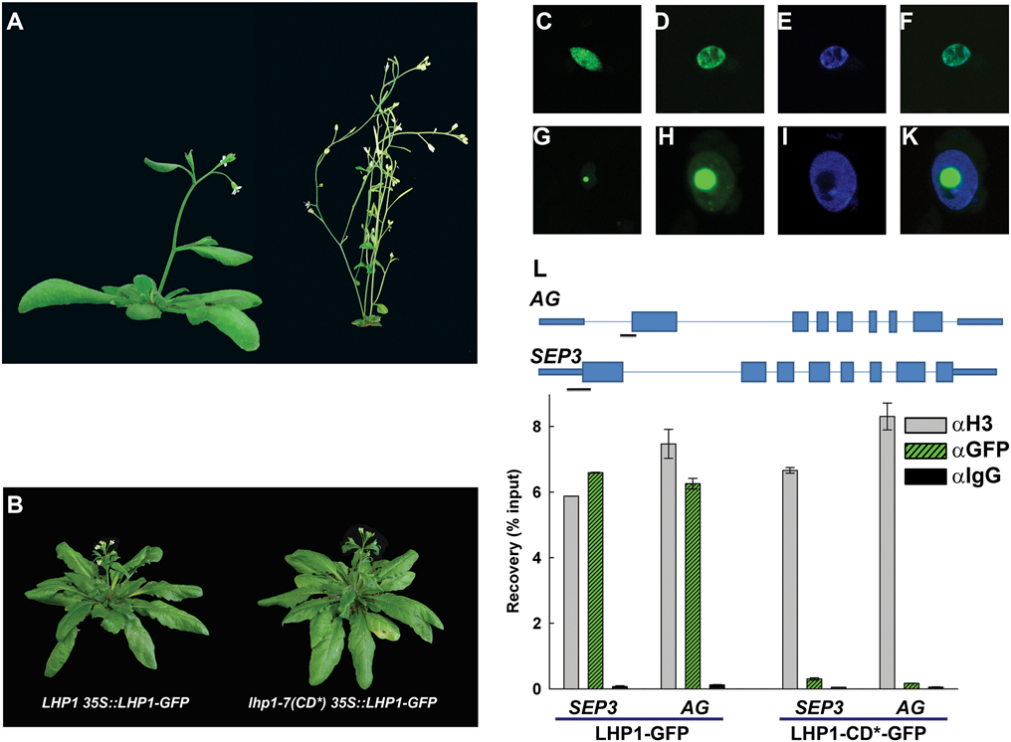
Altered sub-nuclear localization of LHP1-CD*-GFP. (A) Wild-type (Col, left) and lhp1-7(CD*) (right) after five weeks of growth under long day photoperiod. (B) *LHP1 35S::LHP1-GFP* (left) and lhp1-7(CD*) 35S::LHP1-GFP (right) plants. Plants are in the *msi1-tap1* background. (C-K) *35S::LHP1-GFP* (C-F) and 35S::lhp1-7(CD*)-GFP plants (G-K) were used to analyze protein localization in leaf nuclei. Protein localization was detected by confocal laser scanning microscopy of GFP-fluorescence (C, G) or by immuno-localization (D, H). (E, I) DAPI-staining of the nuclei in D and H; merged images of D and E (F) and of H and I (K). (L) ChIP assays for binding of LHP1-GFP and LHP1-CD*-GFP to the *AG* and *SEP3* loci. Top: Genomic structure of *AG* and *SEP3*. Lines represent introns, narrow bars 3′ and 5′ UTRs and wide bars represent coding exons. Black lines represent regions probed by qPCR. Values are recovery as percent of input; IgG served as negative control.

To analyze the *in vivo* activity of LHP1-CD*, we introduced LHP1-GFP and LHP1-CD*-GFP fusion proteins into lhp1-7(CD*). We found several lines in which the LHP1-GFP fusion protein could complement lhp1-7(CD*), demonstrating that LHP1-GFP is fully functional (Fig. 3A, B). In contrast, the LHP1-CD*-GFP fusion protein was expressed (Fig. 3G, H) but unable to complement the mutant, suggesting that LHP1-CD*-GFP cannot substitute for wild-type LHP1.

Microscopic inspection of the LHP1-GFP and LHP1-CD*-GFP lines revealed that both wild-type and the mutant fusion proteins were targeted to the nucleus. The LHP1-GFP fusion protein showed a speckled pattern throughout the nucleus in most lines (Fig. 3C–F), similar to published data [13]. In contrast, the mutant LHP1-CD* was more uniformly distributed in the nucleus, often with additional strong accumulation in the nucleolus (Fig. 3G–K). Accumulation of mutant LHP1 versions in the nucleolus has been reported before [13], [22], but the relevance of this abnormal targeting is unknown.

Altered *in vitro* binding and sub-nuclear distribution of LHP1-CD* could also affect binding to individual target loci. We used the GFP fusion lines to test binding of LHP1 to *AGAMOUS* (*AG*) and *SEPALATA3* (*SEP3*), which are well-established PcG and LHP1 targets [14]–[16], [23]. After chromatin immunoprecipitation we found that LHP1-GFP, but not LHP1-CD*-GFP, bound efficiently to both loci (Fig. 3L). Together, these results show that LHP1-CD* lost specificity for H3K27me3 *in vitro* and that LHP1-CD*-GFP cannot bind to at least some LHP1 targets *in vivo*, which may explain its altered sub-nuclear localization.

### Development is altered in lhp1-7(CD*) mutants

**Figure 4:**
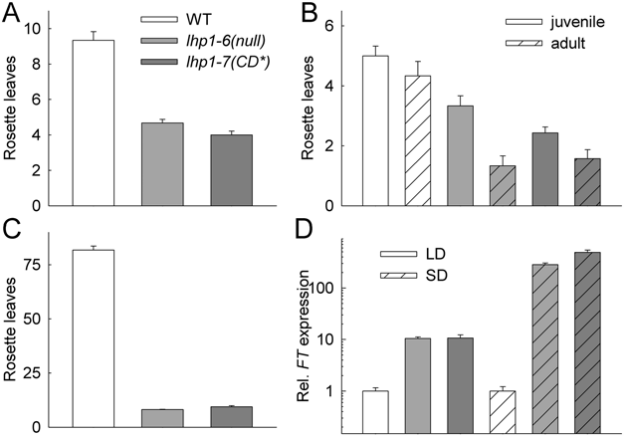
Early flowering of *lhp1* mutants. (A) Rosette leaves produced until bolting in long days (LD). (B) Phase transition in LD. (C) Rosette leaves formed until bolting in SD. Values in (A-C) are mean±S.E. (n≥7). (D) FT expression at ZT = 4h (ZT, *zeitgeber* time; ZT = 0 is lights on) in 12 days old seedlings from LD and at ZT = 6h in 14 days old seedlings from SD. Samples were taken at times when *FT* expression in wild-type is low [43]. Values in D are mean±S.E. (n = 4). Note that expression values for LD and SD were independently normalized to the corresponding wild-type. For all panels: White, grey, and dark-grey bars represent wild-type, *lhp1-6(null)* and lhp1-7(CD*), respectively.

**Figure 5:**
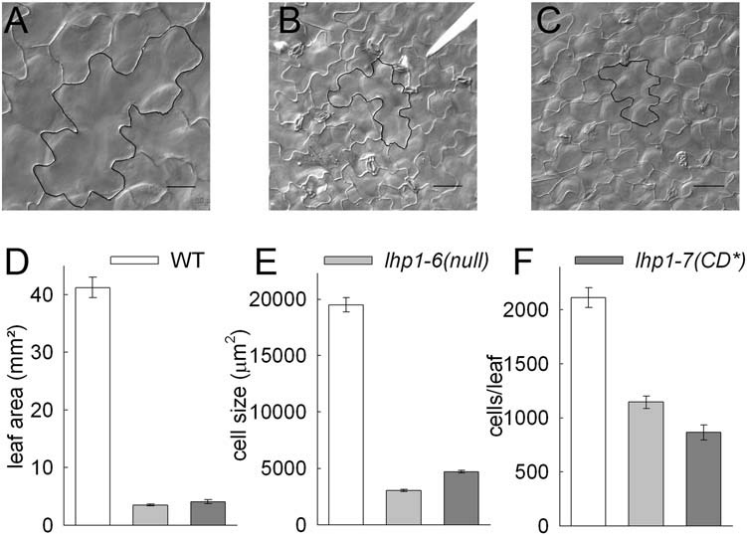
Altered leaf development in *lhp1* mutants. (A-C) Adaxial epidermis of Col (A), *lhp1-6(null)* (B) and lhp1-7(CD*) (C) leaves. (D) Area of first and second rosette leaf after bolting (n≥11). (E) Cell size in the adaxial epidermis of the first and second rosette leaves (n≥244). (F) Estimated cell number in the adaxial epidermis of the first and second rosette leaf. For all panels: White, grey, and dark-grey bars represent wild-type, *lhp1-6(null)* and lhp1-7(CD*), respectively. Values in (D-F) are mean±S.E.

We compared the lhp1-7(CD*) mutant to wild-type and *lhp1-6*(null) mutant plants to establish which aspects of LHP1 function depend on chromodomain binding to H3K27me3. Analysis of flowering time revealed that both lhp1-7(CD*) and *lhp1-6(null)* plants flowered at similar times but much earlier than wild-type under long and short day conditions (Fig. 4). Early flowering was characterized by shortened juvenile and adult phases concomitant with strong *FT* upregulation (Fig. 4B, D). Epidermal cells of *lhp1* mutant rosette leaves were much smaller, although they maintained the characteristic jigsaw like shape (Fig. 5). Leaf cell number and expansion were reduced in both *lhp1* alleles, causing a strongly decreased rosette leaf size (Fig. 5).

**Figure 6:**
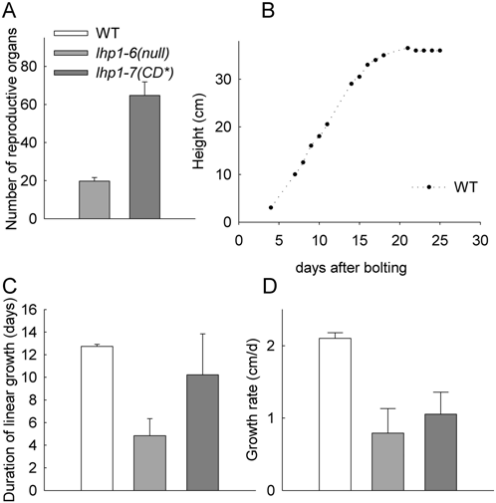
Altered shoot development in lhp1 mutants. (A) The total number of reproductive organs (siliques, flowers and flower buds) on the primary shoots of five weeks old plants from LD. Values are mean±S.E. (n≥8). (B) Example of the growth curve of a wild-type plant's primary shoot. (C) Length of linear growth phase. (D) Growth rates during linear growth phase of primary shoots. Values in (C, D) are averages over two experiments with n≥9 per experiment. For all panels: White, grey, and dark-grey bars represent wild-type, *lhp1-6(null)* and lhp1-7(CD*), respectively.

Arabidopsis LHP1 was initially identified genetically for its terminal flower phenotype [10]. Both *lhp1-6*(null) and lhp1-7(CD*) have the terminal flower phenotype, but lhp1-7(CD*) formed the terminal flower later than *lhp1-6(null)* (Fig. 6A). Consistently, primary stem growth ceased much earlier in *lhp1* mutants than in wild-type plants, but later in lhp1-7(CD*) than in *lhp1-6(null)* (Fig. 6B, C). In both *lhp1* alleles, not only duration of primary stem growth but also growth rates were reduced (Fig. 6D). Together, lhp1-7(CD*) is phenotypically similar to *lhp1-6(null)* during early plant development, but has a slightly milder phenotype late in development.

### Silencing of PcG target genes is lost in lhp1-7(CD*) mutants

**Figure 7:**
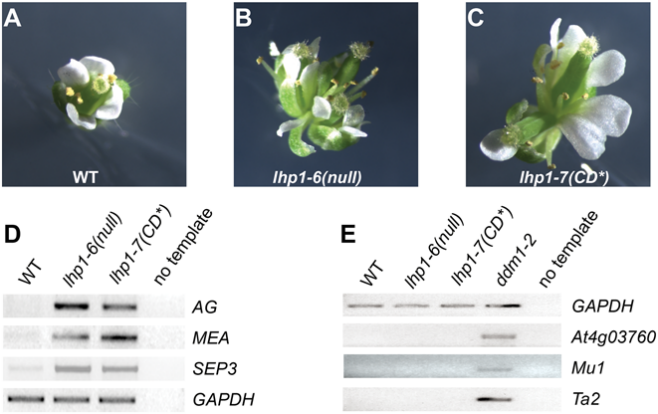
Loss of silencing at PcG targets and maintenance of silencing at heterochromatic loci in lhp1 mutants. (A–C) Flowers of wild-type Col (A), *lhp1-6(null)* (B) and lhp1-7(CD*) (C) produced late during development. (D) Expression of PcG targets in seedlings at ZT = 5h after 16 days in LD. (E) Expression of heterochromatic loci in rosette leaves at ZT = 5h after 25 days in LD. RNA from *ddm1-2* was used as positive control.

Flowers produced late during *lhp1-6* and lhp1-7(CD*) development often have supernumerary, missing or deformed organs (Fig. 7A–C), which may be caused by deregulation of floral homeotic genes. *AG* and *SEP3* were ectopically expressed in *lhp1-6(null)* and lhp1-7(CD*) rosette leaves (Fig. 7D). Similarly, *MEDEA* and *AGL19*, two PcG targets [24], [25], were de-repressed in both *lhp1* alleles (Fig. 7D and data not shown). The observation that there was no reactivation of transposons or pseudogenes (*At4g03760*, *MU1*, *TA2*) or of targets of the RNA-dependent DNA-methylation pathway (*IG/LINE*, *IG2*, *IG5*, *RPL18*) (Fig. 7E and data not shown) confirmed that loss of LHP1 does not affect silencing in heterochromatin [13], [14].

Together, our results show that similar to *lhp1-6(null)* major developmental regulatory genes (e.g., *FT*, *AG* and *SEP3*) are not repressed in lhp1-7(CD*) at times when they should be silent. Thus, we conclude that specific binding of LHP1 to H3K27me3 is essential to maintain repression of PcG target genes.

## Discussion

**Figure 8:**
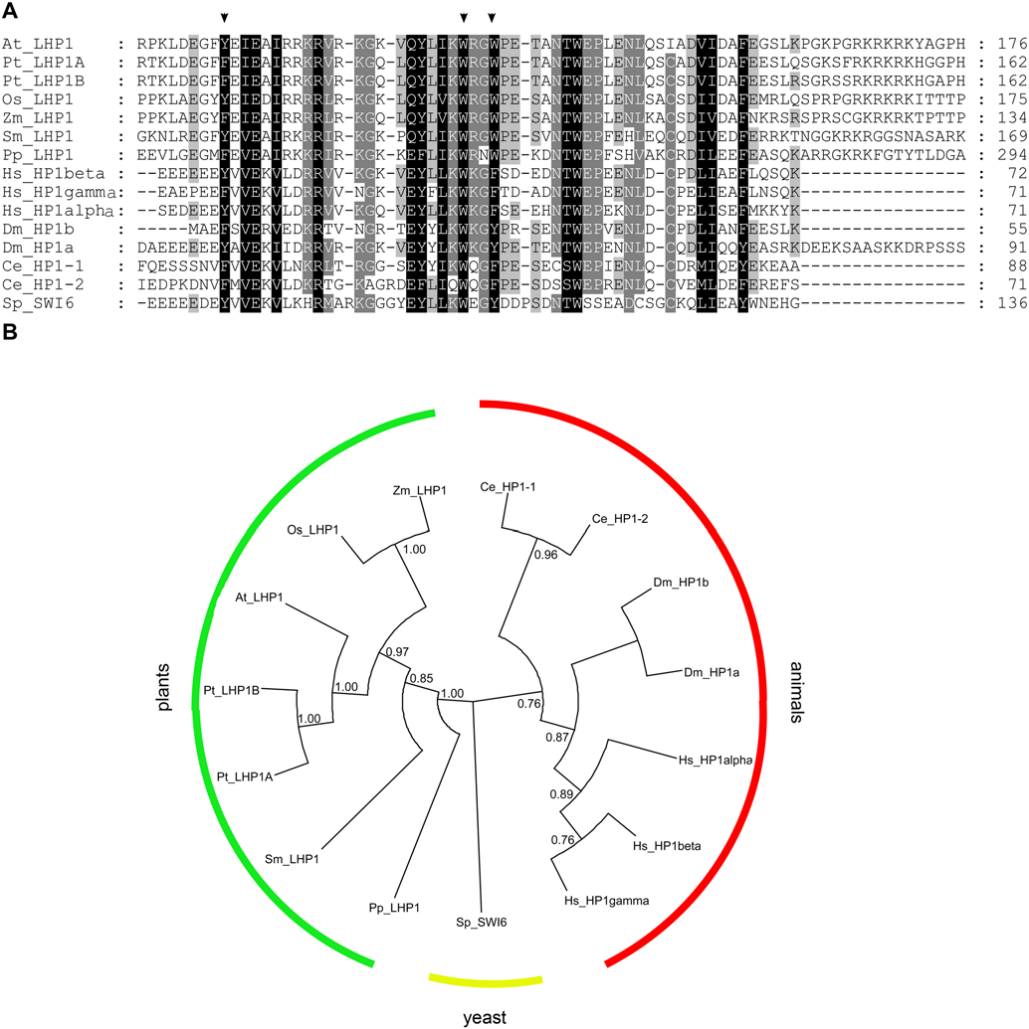
Sequence alignment and phylogenetic tree of LHP1 and HP1 homologues. (A) Segment of the alignment that contains the chromodomain. The arrow heads highlight the aromatic cage residues that form the binding cavity for histone methyl groups. (B) Phylogenetic tree of 15 LHP1 and HP1 homologs (*Arabidopsis thaliana*: At_LHP1 GI:15625407. *Populus trichocarpa*: Pt_LHP1A, estExt_Genewise1_v1.C_LG_XIX1329; Pt_LHP1B, eugene3.00130688. *Oryza sativa*: Os_LHP1, GI:110810411. *Zea mays*: Zm_LHP1 GI:22135459. *Physcomitrella patens*: Pp_LHP1, jgi|Phypa1_1|169812|estExt_fgenesh1_pg.C_2200058. *Selaginella moellendorffii*: Sm_LHP1, jgi|Selmo1|407083|fgenesh2_pg.C_scaffold_6000334. *Homo sapiens*: Hs_HP1alpha, GI:6912292; Hs_HP1beta, GI:48428808; Hs_HP1gamma, GI:5732187. *Drosophila melanogaster*: Dm_HP1a, GI:17136528; Dm_HP1b, GI:24640713. *Caenorhabditis elegans*: Ce_HP1-1, GI:17568757; Ce_HP1-2, GI:71987888. *Schizosaccharomyces pombe*: Sp_SWI6, GI:510930.). The evolutionary history was inferred using the Maximum Parsimony method; the most parsimonious tree with length = 2089 is shown. Support for each node, assessed with bootstrap analysis (1000 replicates) is given when higher than 60%. Note that the tree is displayed as circular cladogram with all branches of the same length.

In animals, PRC2 complexes set H3K27me3 marks, which assist to recruit PRC1 to mediate stable silencing [2]. Plant LHP1 proteins are similar to metazoan HP1, but could have PRC1 functions. Phylogenetic analysis suggests that the LHP1 and HP1 protein subfamilies have strongly diverged (Fig. 8). In addition to Arabidopsis, genes for LHP1 homologues were previously described for multiple mono- and dicotyledonous plant species such as apple, rape seed, carrot, tomato, rice and maize [11], [12], [26]. We found LHP1 homologues also in the genomes of poplar (*Populus trichocarpa),* of a lycophyte (*Selaginella moellendorffii*), an ancient vascular plant lineage, and of a moss (*Physcomitrella patens*). In contrast, we failed to identify LHP1 or HP1 homologs in the genomes of the chlorophyte algae *Volvox carteri* and *Chlamydomonas reinhardtii*, suggesting that the presence of LHP1 is linked to multicellular development in the plant kingdom. Because chromatin immunoprecipitation has shown that LHP1 binding overlaps with H3K27me3 and LHP1 can bind H3K27me3 *in vitro*, it was suggested that the chromodomain-protein LHP1 is a PRC1 equivalent of plants [15], [16]. In contrast to animals, however, where PRC1 is needed for spreading of H3K27me3 over extended regions, in plants loss of LHP1 does not affect genomic H3K27me3 distribution [15].

Three aromatic residues form the binding cavity for methylated lysines of H3 in the chromodomain of animal HP1 and Pc [3], [20], [21], [27]. Based on protein homology modeling, the chromodomain of plant LHP1 forms a similar binding pocket. Therefore we suggest that the novel lhp1 allele lhp1-7(CD*) has a defective binding pocket for the quaternary ammonium group because the preference of LHP1 for H3K27me3 over H3K27 was lost for LHP1-CD*. Energy calculations using CHARMM [28] and the CHARMm [29] force field are in qualitative agreement with the relative affinities measured by the pull-down assay. A quantitative agreement is not expected because of approximations inherent to the force field and the qualitative nature of the pull-down assays. An LHP1-CD*-GFP fusion did not efficiently bind to target gene chromatin and had lost its correct sub-nuclear distribution, suggesting that chromodomain-mediated binding to H3K27me3 is essential for LHP1 targeting *in vivo*. In contrast, the chromodomain might not be necessary for targeting of animal HP1 *in vivo* [30]–[32].

Mutations in Arabidopsis LHP1 strongly affect development [10], [12]. The phenotype of the lhp1-7(CD*) allele was very similar to that of an *lhp1* null allele, suggesting that LHP1 function requires an intact chromodomain. Because only LHP1-GFP but not LHP1-CD*-GFP could rescue *lhp1* mutants, LHP1-CD* has no or strongly reduced biological activity. Residual binding of LHP1-CD* to H3K27me3 could explain the phenotypic differences between lhp-7(CD*) and *lhp1-6(null)* plants.

Loss of LHP1 or PRC2 share many similar developmental and molecular effects. Our experimental results, supported by homology modeling and previous reports, have revealed that LHP1 contributes to PRC1-like functions in plants and that chromodomain-mediated binding to H3K27me3 is required for this activity.

## Materials and Methods

### Plant material and growth conditions

All mutants used are in the Columbia (Col) wild-type accession of *Arabidopsis thaliana*. The *ddm1-2* allele was described before [33]. A new *lhp1* allele, *lhp1-6*, was identified in the SALK T-DNA insertion mutant collection (SALK_011762). *LHP1* and *lhp1-7* cDNAs were cloned into vector pK7FWG2 [34], which was used to transform plants by floral dip with *Agrobacterium tumefaciens* (strain GV3101). Seeds were germinated on sterile basal salts Murashige and Skoog (MS) medium (Duchefa, Brussels, Belgium), and plants were analyzed on plates or transferred to soil 10 days after germination. Alternatively, seeds were directly sown on soil. Plants were kept in Conviron growth chambers with mixed cold fluorescent and incandescent light (110 to 140 µmol m^−2^ s^−1^, 21±2°C) under long day (LD, 16h light) or short day (SD, 8h light) photoperiods or were alternatively raised in green houses.

### Isolation of the new lhp1-7(CD*) allele

For a suppressor screen, seeds of the late flowering *msi1-tap1* transgenic line [18] were mutagenised with ethyl methane sulfonate (EMS). Approximately one thousand F2 families were screened for suppression of the delayed floral transition of *msi1-tap1*. One family (0.3 362) segregated plants with a conspicuous early flowering phenotype. These early flowering plants were smaller, had reduced fertility and segregated in a 1:3 ratio (data not shown), suggesting recessive Mendelian inheritance. Molecular mapping located the mutation between the markers CER456657 (BAC MPI7) and CER457604 (BAC MXE10) on the top arm of chromosome V. Within the same region lies the gene *At5g17690*, which encodes LHP1. Because of similarities between the phenotypes of 0.3 362 plants and *lhp1* mutants, the *At5g17690* locus in 0.3 362 was sequenced and a single G to A transition was discovered.

To confirm that the mutation in the LHP1 gene is indeed responsible for the observed phenotype, an allelism test between 0.3 362 and the *lhp1-6* null allele was performed. The analyzed F1 and F2 generations displayed a homogenous appearance with small rosette size and were early flowering (data not shown), while genotyping revealed the expected ratios of plants homozygous, heterozygous or negative for the presence of the *lhp1-6* T-DNA insertion (data not shown), confirming that 0.3 362 was allelic to *lhp1-6*. The newly identified *lhp1* allele was henceforth called *lhp1-7*.

### Flowering time and growth kinetics

Flowering time was defined as the time needed by the plants (n>7) to form a 5 mm high primary shoot. In addition, the numbers of juvenile and adult rosette leaves were determined based on the presence of abaxial trichomes as indicators for phase identity [35]. For growth kinetics, the height of the primary shoot was measured daily. The end of the linear growth phase was determined manually for individual plants from height vs. time after bolting diagrams. Primary shoots of wild-type plants grew linearly for nearly two weeks after bolting before growth ceased gradually (Fig. 6B).

### *In vitro* transcription/translation and pull down assays

*LHP1* and *lhp1-7* cDNAs were cloned into vector pRSET-A (Invitrogen) for *in vitro* transcription/translation reactions (TNT® T7 Quick Coupled Transcription/Translation System, Promega, Madison, WI) supplemented with L-[^35^S]methionine. Equal amounts of wild-type and mutant protein were incubated with H3K27 or H3K27me3 peptides (LATKAARKSAPATGGC) coupled to SulfoLink Coupling Gel (Pierce Perbio, Lausanne, Switzerland). Samples were resolved by SDS-PAGE, exposed to a storage phosphor screen (Amersham Biosciences, Otelfingen, Switzerland) and visualized using a Molecular Imager FX Pro Plus System (BioRad, Reinach, Switzerland).

### RNA isolation, RT-PCR and Real Time PCR

RNA isolation and RT-PCR was performed as previously described [36]. For Q-PCR analysis, the Universal Probe Library system (Roche Diagnostics, Rotkreuz, Switzerland) was used on a 7500 Fast Real-Time PCR instrument (Applied Biosystems, Lincoln, CA). *PP2A* was used as reference gene [37]. Q-PCR was performed with three to four replicates, and results were analyzed as described [38]. For details of the assays see Table 2.

**Table 2:** Assays used for qPCR.

Gene	Forward primer	Reverse primer	Universal Probe Library probe
*FT*	GGTGGAGAAGACCTCAGGAA	GGTTGCTAGGACTTGGAACATC	#138 (Arabidopsis)
*PP2A*	GGAGAGTGACTTGGTTGAGCA	CATTCACCAGCTGAAAGTCG	#82 (Arabidopsis)
*AG*	CTAATCAAATTTTGCCCTAAACG	TCCTAGCTCCGATTGGTACG	#132 (Arabidopsis)
*SEP3*	ATTGATCTTGTTCTCTATCCTCTTCAA	AGAGAGAGAGATTGAGATATCTTTTGG	#103 (Arabidopsis)

**Table 3:** Primer sequences.

Primer-ID	Sequence
LH156	TGCATATTTGCGCTTCCGTTT
LH157	CGGTGGAAACAGTCGGAGAAA
LH369	GGAAGGCTAGAGTTGTTGAGAGAC
LH473	GGTTCAGTATCTAATTAAATGTTGTGAAAG
LH474	GGCAAGGTTCAGTATCTAATTAAATGG

### Immuno-localisation

Immuno-localization of GFP fusion proteins was performed as described previously [39] using nuclei isolated from rosette and cauline leaves and a rabbit anti-GFP antibody (A11122, Molecular Probes Invitrogen, Basle, Switzerland). For detection, Alexa Fluor® 488 goat anti-rabbit IgG (A11008; Molecular Probes Invitrogen, Basle, Switzerland) was used. The preparations were analyzed by either epifluorescence microscopy (Zeiss Axioplan 2) or by confocal laser microscopy (Leica TCS SP1). For confocal laser microscopy, Alexa fluorophores were excited with a 488 nm laser; the emission signal was collected in a wavelength window between 502 nm and 543 nm. DAPI fluorescence was collected in a window from 438–485 nm

### Structure determination by homology modeling

Wild-type and mutant sequences were processed by the SwissModel server [40] in automatic mode, fixing as a template the A chain of 1PDQ (Drosophila Polycomb chromodomain complexed with the histone H3 tail containing trimethyl lysine 27 [3]). CHARMm atom types and force field parameters [29] were assigned for all structures. Hydrogen atoms were added and minimized with the program CHARMM [28]. Trimethylated and unmethylated lysine residues were blocked with acetyl and N-methyl-aminyl groups. They were then minimized in the rigid protein using CHARMM and a distance-dependent dielectric function (ε = 4r). During minimization, harmonic constraints with a force constant of 2.5 Kcal/mol/Å2 were added to the blocking groups. The starting position of the trymethylated lysine residue was obtained by superimposing 1PDQ to each model. The interaction energy between the protein and the trymethylated/unmethylated lysine residue was calculated by INTE command of CHARMM. Given the approximations inherent to the force field and the homology models, only a qualitative agreement with experimental data is expected.

### Chromatin Immunoprecipitation

Chromatin isolation was performed as described previously [24] using 15d-old seedlings. Chromatin immunoprecipitation was done using the LowCell# ChIP kit (Diagenode, Liège, Belgium) according to manufacturer's instructions. The following antibodies were used: Polyclonal anti-H3 antibody (#01-690, Upstate, Charlottesville, VA), polyclonal anti-GFP antibody (#A11122, Molecular Probes Invitrogen, Basle, Switzerland) and non-immun IgG (Diagenode). Presence of *AG* and *SEP3* fragments was determined by qPCR using the Universal Probe system (Roche).

### Sequence alignment and phylogenetic analysis

Protein sequences of HP1 and LHP1 proteins were selected based on previous publications [16], [26] and on BLAST searches with the Arabidopsis LHP1 sequence using the DOE Joint Genome Institute data base (http://genome.jgi-psf.org/). Final sequence alignments of the selected sequences were generated with CLUSTALX 1.81 (protein weight matrix was Gonnet 250, gap opening penalty was 10.0, and gap extension penalty was 0.2). The evolutionary history was inferred using the flat-weighted Maximum Parsimony method. The MP tree of amino acid sequences was obtained using the Close-Neighbor-Interchange algorithm with search level 3 in which the initial trees were obtained with the random addition of sequences (10 replicates). All alignment gaps were treated as missing data. There were a total of 985 positions in the final dataset, out of which 292 were parsimony informative. Phylogenetic analyses were conducted in MEGA4 [41]. The presentation of the phylogenic tree was prepared using Dendroscope [42].
